# Comparison Between Effect of Preoperative Verbal Counseling Versus Preoperative Counseling Using Anesthesia Information Sheet on Anxiety of Patients Undergoing Elective Surgeries: A Randomized Comparative Study

**DOI:** 10.7759/cureus.64667

**Published:** 2024-07-16

**Authors:** Aparna Bagle, Mounika Yerramshetty, Ishan G Garud

**Affiliations:** 1 Anesthesiology, Dr. D. Y. Patil Medical College, Hospital and Research Centre, Pune, IND

**Keywords:** pre-operative anxiety, patient counseling, pre-operative counseling, non-pharmacological intervention, perioperative anxiety, hamilton anxiety scale

## Abstract

Introduction

Preoperative anxiety refers to the feelings of unease, fear, or nervousness experienced by individuals before undergoing a surgical procedure. This anxiety can stem from various sources, including fear of the unknown, concerns about the surgical outcome, worries about pain or complications, and separation from loved ones during the procedure. Healthcare professionals can help minimize preoperative anxiety by employing various strategies and promoting better surgical experiences and outcomes. Hence, this study was designed to compare the effect of conventional preoperative verbal counseling versus preoperative verbal counseling using an anesthesia information sheet (AIS) on pre‑operative anxiety of patients.

Methods

A total of 80 patients were randomly placed into two groups of 40 each - preoperative verbal counseling (PC) and verbal counseling using an AIS. The Hamilton Anxiety Scale (HAM-A) was used to assess preoperative anxiety in both the group's pre- and post-counseling. Data was collected and compiled. Data was analyzed using SSPS software. Pearson correlation coefficient was used for the correlation of age, gender, education status, and ASA grading with pre- and post-counseling anxiety scores.

Results

A significant difference was seen in anxiety score pre- and post-counseling between group PC and AIS (p-value <0.05). The anxiety score after counseling in group PC was 16.27±4.57, which was significantly higher compared to group AIS (14.25±2.42; p-value=0.016).

Conclusion

As we continue to explore innovative ways to improve patient experiences and outcomes, integrating AISs into counseling practices stands as a promising strategy that can lead to more confident and well-informed patients, ultimately enhancing the quality of healthcare delivery.

## Introduction

Preoperative anxiety, defined as the apprehension or fear experienced by individuals prior to undergoing surgical procedures, encompasses a range of emotional responses that can significantly influence patient outcomes. This emotional state often arises from uncertainties surrounding the surgical process, including concerns about pain, surgical outcomes, and the effects of anesthesia. Its prevalence underscores its relevance in clinical practice, impacting patient comfort, surgical performance, and recovery trajectories. Recognizing and addressing preoperative anxiety is critical for optimizing perioperative care and enhancing patient satisfaction, thereby warranting attention from both anesthetists and surgeons in comprehensive patient management protocols. According to the results of an observational study of more than 15000 patients undergoing a non-obstetric surgical procedure, anxiety was most frequently mentioned to be the worst aspect of the perioperative period [1]. Various studies have examined anxiety-provoking aspects underlying preoperative anxiety, with only some of these studies reporting intensities of concern and few of these focusing on potential adverse events and complications, particularly associated with anesthesia [2-5]. Pharmacological medications are used to reduce anxiety, but they may induce some adverse effects.

In contrast, various non-pharmacological interventions, such as reassurance, music therapy, breathing exercises, meditation, acupressure, and pre-procedure education, are used to allay preoperative anxiety. These approaches are inexpensive, easy to perform, do not require a high level of technical skill or equipment, and are without adverse effects [6]. Only one study undertaken this far has used an anesthesia information sheet (AIS), which evaluated anxiety using the Visual Analog Scale (VAS-A). The Hamilton Anxiety Scale (HAM-A) was one of the first rating scales developed to measure the severity of anxiety symptoms and is still widely used today in clinical and research settings. The scale consists of 14 items, each defined by a series of symptoms, and measures both psychic anxiety (mental agitation and psychological distress) and somatic anxiety (physical complaints related to anxiety) [7]. Hence, this study was designed to compare the effects of different types of counseling using the HAM-A, which is a widely used scale for assessing anxiety.

## Materials and methods

The study was conducted in Dr. D.Y. Patil Medical College, Hospital and Research Centre, Pune, India. After receiving the approval of the institutional ethics committee (I.E.S.C/89/2023), this prospective randomized comparative study was started.

The inclusion criteria consisted of American Society of Anesthesiologists (ASA) grade I, II, or III patients, aged between 18 to 65 years female and male patients, patients able to read at least one language Marathi, Hindi, or English, and patients posted for elective surgeries and availability of informed consent.

The exclusion criteria consisted of patients with ASA physical status IV or more, patients who are not giving consent to the above study, patients below 18 years and above 65 years of age, and patients with any psychological diseases or on antidepressant/antianxiety medications.

**Figure 1:**
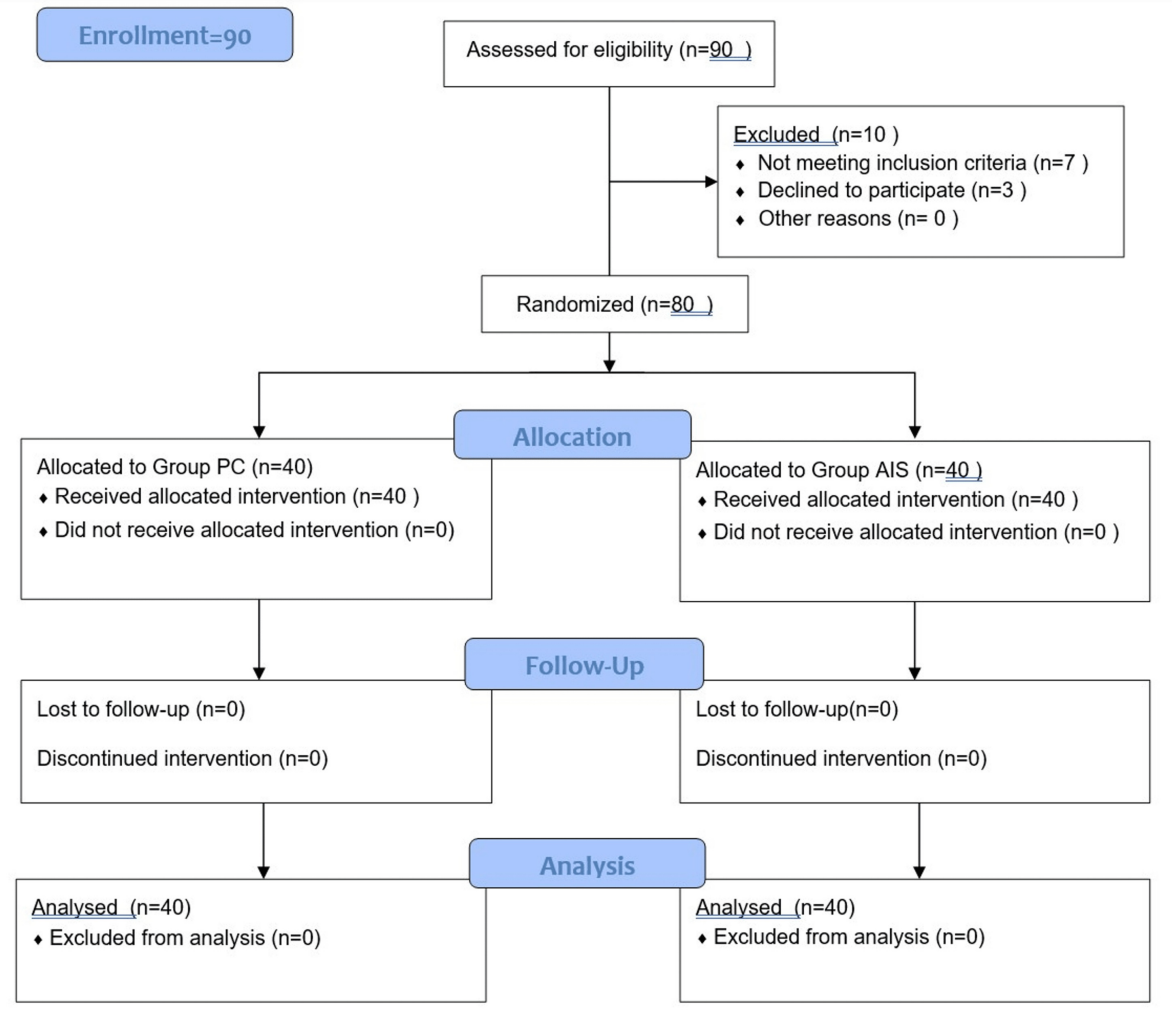
CONSORT flow diagram AIS: anesthesia information sheet; PC: preoperative verbal counseling

The sample size for this study was calculated using WINPEPI software with a confidence interval of 95% and power of study of 80%. We needed a total sample size of 54 but for better accuracy, we took a sample size of 80, 40 in each group as seen in Figure 1.

After obtaining a written informed consent, the patients were allocated randomly into two groups. A comprehensive AIS was developed in English and Hindi, supplemented with illustrative diagrams demonstrating various anesthesia administration techniques. The document provided detailed explanations of the concept of anesthesia, the available modalities, and their advantages and disadvantages. It also covered essential aspects such as patient preparation before anesthesia administration and guidelines for postoperative care.

On the day prior to the surgery, patients were randomly allocated into two groups: 1) preoperative verbal counseling (PC) and 2) preoperative counseling using an AIS (Figures 10, 11 of Appendices). Each patient's anxiety level was assessed using the HAM-A in which each item is scored on a scale from zero (not present) to four (severe), with a total severity range of 0-56, where <17 indicates mild, 18-24 indicates mild to moderate, 25-30 indicates moderate to severe, and >30 indicates severe anxiety. The patients were asked about their history and the possible cause of anxiety to gain an insight into the cause, their education, their income, and any previous negative experiences with anesthesia. The participants were counseled appropriately according to their group allocation. Patient anxiety levels were reassessed by the same investigator two hours later. Participants were stratified into distinct age cohorts with uniform interval distribution.

Statistical analysis

Data was collected and compiled. The quantitative data was analyzed using a t-test or Mann-Whitney test as an appropriate test. The qualitative data was analyzed using a chi-square test. A p-value less than 0.05 was considered statistically significant.

## Results

A total of 80 patients were included in the study, with 40 patients allocated to each group (PC and AIS) as shown in Figure 1. Before counseling, there was no significant difference observed in anxiety scores between the groups (p=0.911). The mean and standard deviation (SD) of the anxiety scores before counseling was 19.8±3.23 for group PC and 19.88±2.73 for group AIS, demonstrating comparable levels of anxiety between the groups. Following counseling, a significant difference emerged in the anxiety scores between the groups (p<0.05). The mean and SD of the anxiety scores after counseling were 16.27±4.57 for group PC, significantly higher than group AIS, measured at 14.25±2.42 (p=0.016), as detailed in Table 1.

**Table 1:** Measures of anxiety in patients pre- and post-counseling ^‡ ^independent t-test;^ § ^paired t-test AIS: anesthesia information sheet; PC: preoperative verbal counseling

Anxiety score	Group PC (n=40)	Group AIS (n=40)	Total	P-value
Pre-counseling				
Mean±SD	19.8±3.23	19.88±2.73	19.84±2.97	0.911^‡^
Median (25th-75th percentile)	19 (17.75-22)	20 (18-21.25)	19.5 (18-22)	
Range	14-28	15-26	14-28	
Post-counseling				
Mean±SD	16.27±4.57	14.25±2.42	15.26±3.77	0.016^‡^
Median (25th-75th percentile)	15.5 (13-20.25)	13.5 (12-16.25)	14 (13-18)	
Range	8-25	10-18	8-25	
Intragroup p-value	<0.0001^§^	<0.0001^§^	-	-

**Figure 2:**
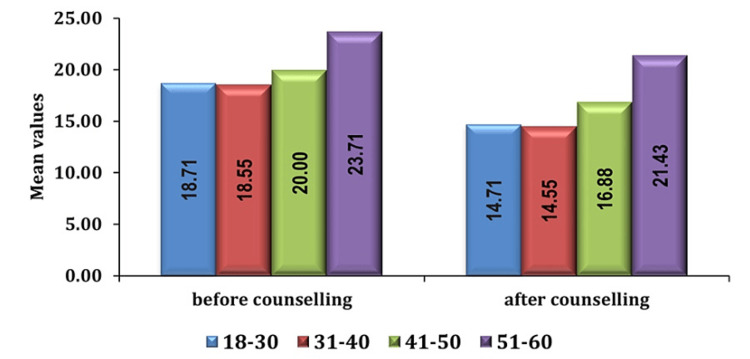
Association of anxiety scores in age group (in years) in group PC pre- and post-counseling PC: preoperative verbal counseling

**Figure 3:**
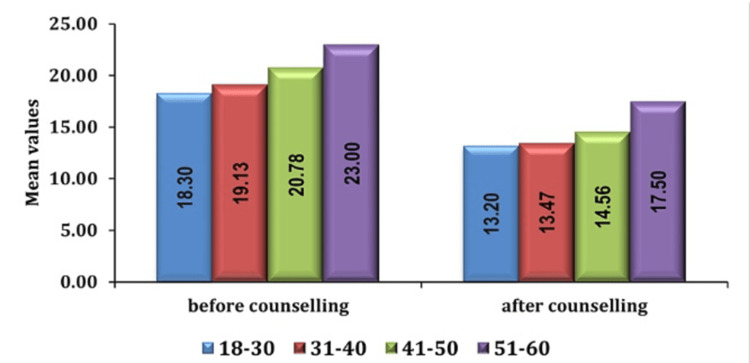
Association of anxiety scores in age groups (in years) in group AIS pre- and post-counseling AIS: anesthesia information sheet

In our study, analysis of demographic variables revealed notable patterns in preoperative anxiety levels among patients. Among the demographic factors, considered age emerged as a significant predictor with elderly individuals, particularly those aged 51-60 years, exhibiting higher levels of preoperative anxiety compared to their younger counterparts as seen in Figures 2, 3.

**Figure 4:**
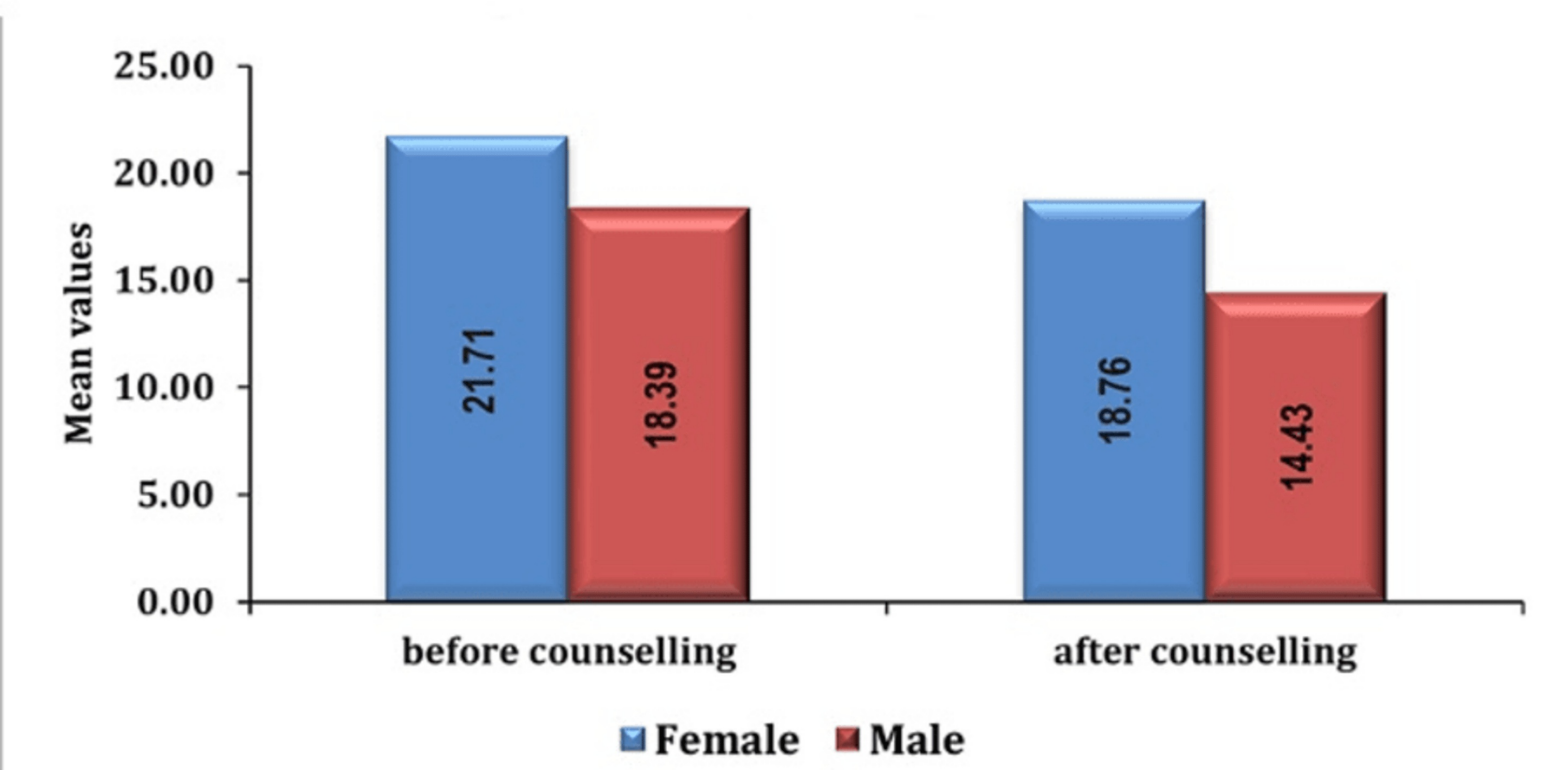
Association of anxiety score with gender in group PC pre- and post-counseling PC: preoperative verbal counseling

**Figure 5:**
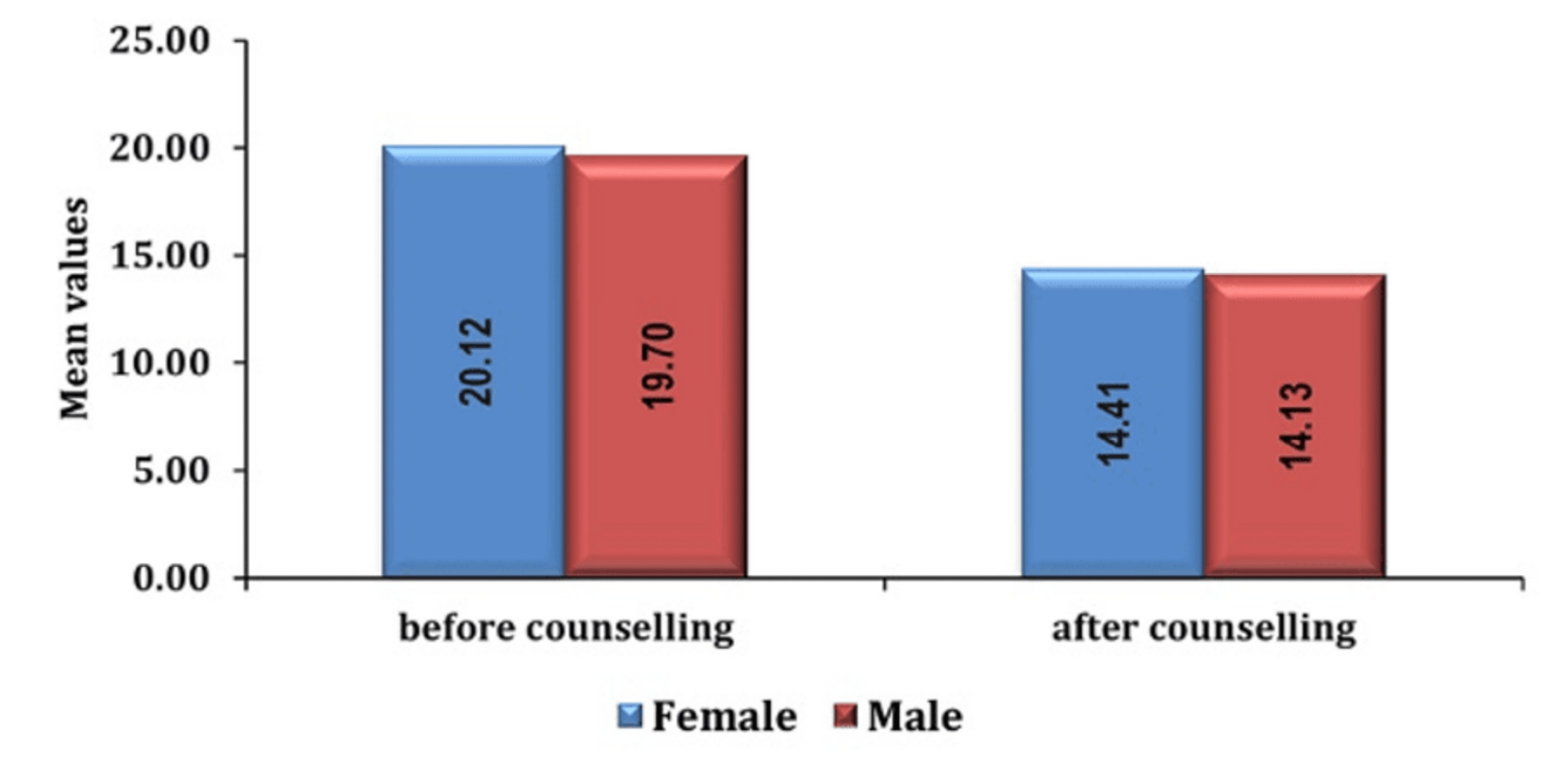
Association of anxiety score with gender in group AIS pre- and post-counseling AIS: anesthesia information sheet

Furthermore, gender differences were observed, with female patients demonstrating elevated preoperative anxiety levels relative to male patients as seen in Figures 4, 5.

**Figure 6:**
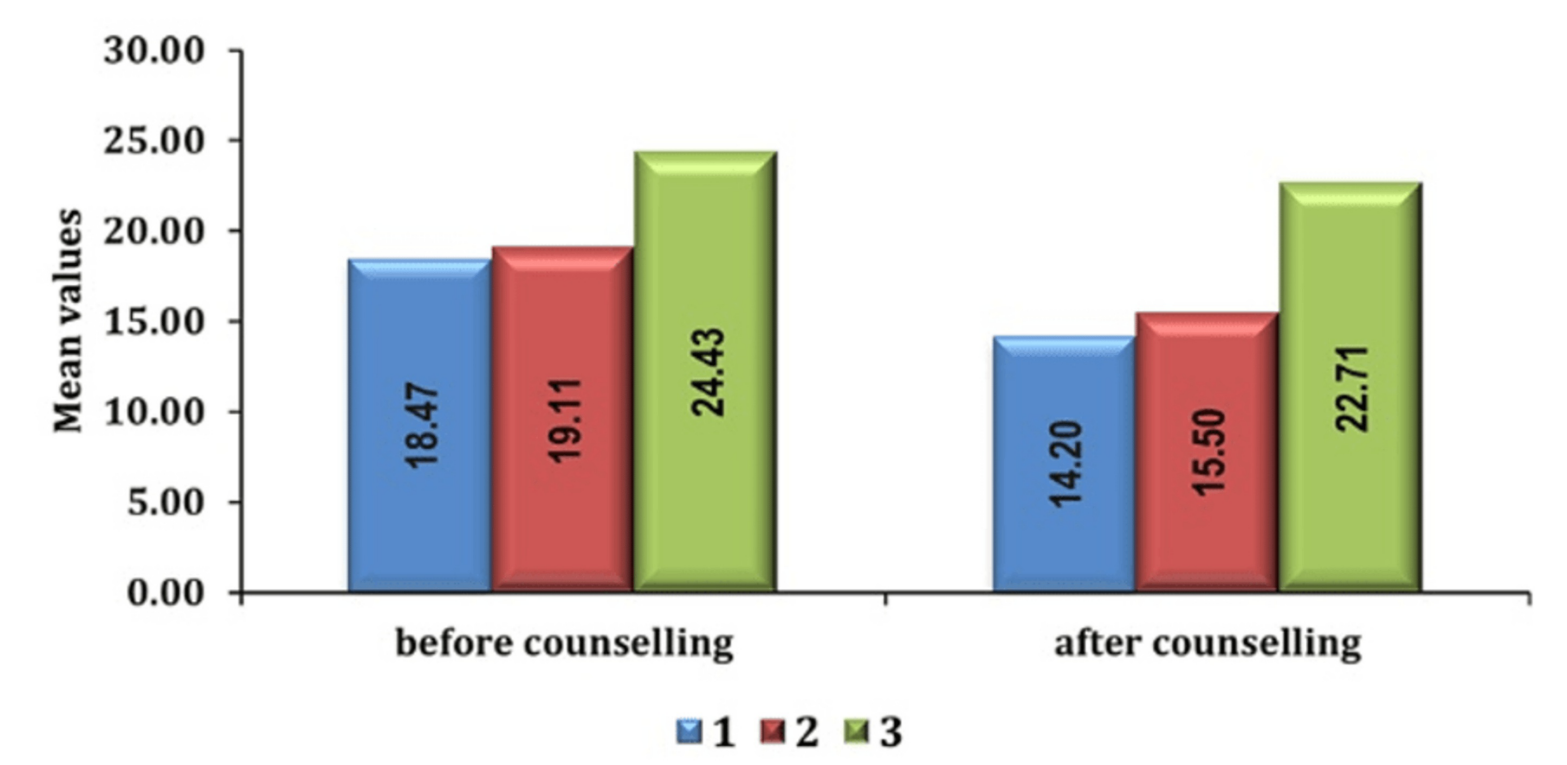
Association of anxiety score with ASA grade in group PC pre- and post-counseling PC: preoperative verbal counseling; ASA: American Society of Anesthesiologists

**Figure 7:**
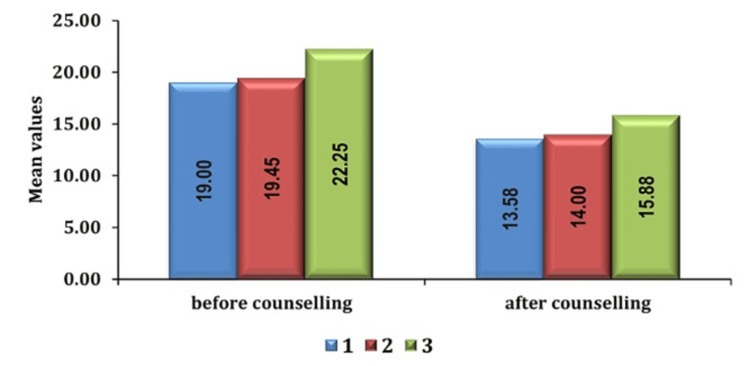
Association of anxiety scores with ASA grade in group AIS pre- and post-counseling ASA: American Society of Anesthesiologists; AIS: anesthesia information sheet

Additionally, patients graded with higher ASA status displayed increased preoperative anxiety levels relative to those with lower ASA status, indicating a correlation between medical condition severity and anxiety levels as seen in Figures 6, 7.

**Figure 8:**
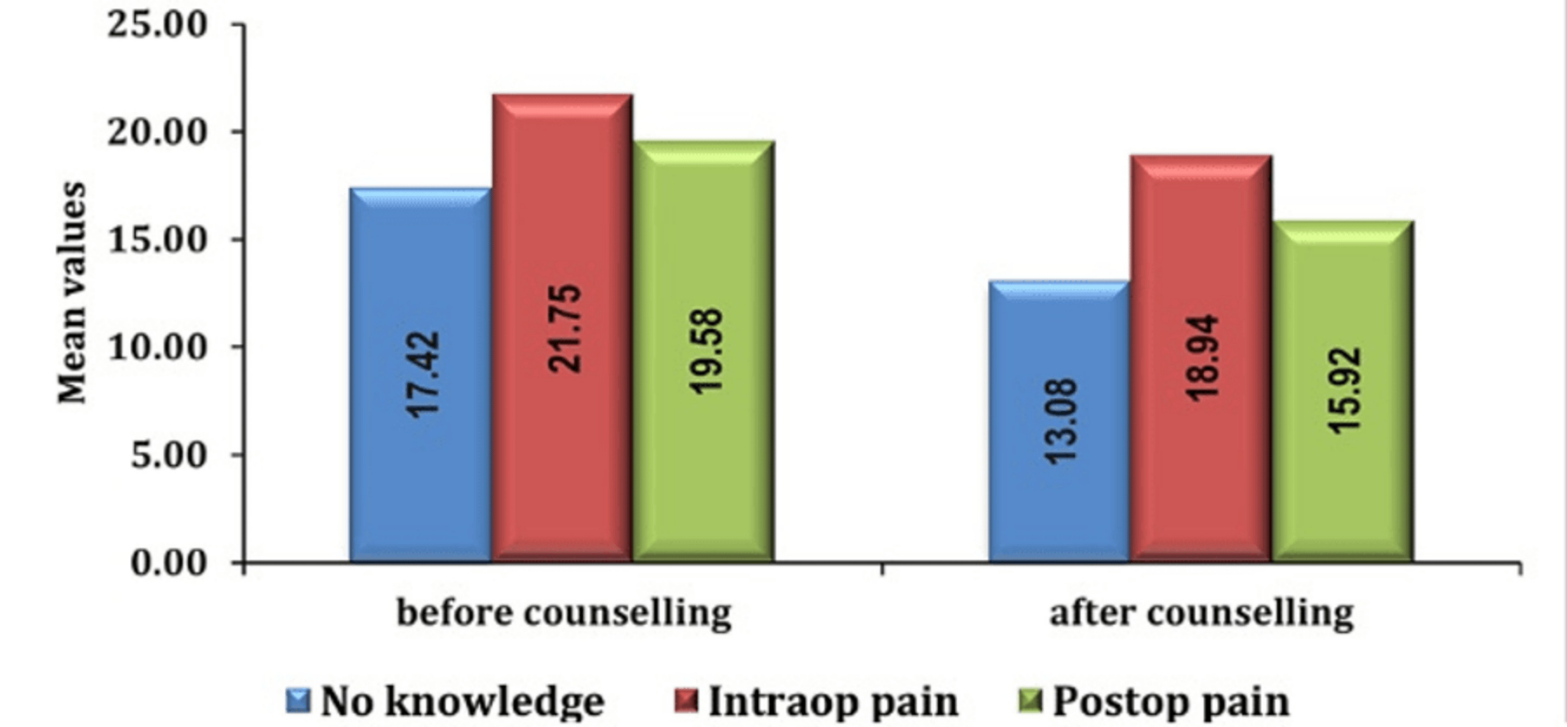
Association of anxiety score and cause of fear in group PC pre- and post-counseling PC: preoperative verbal counseling

**Figure 9:**
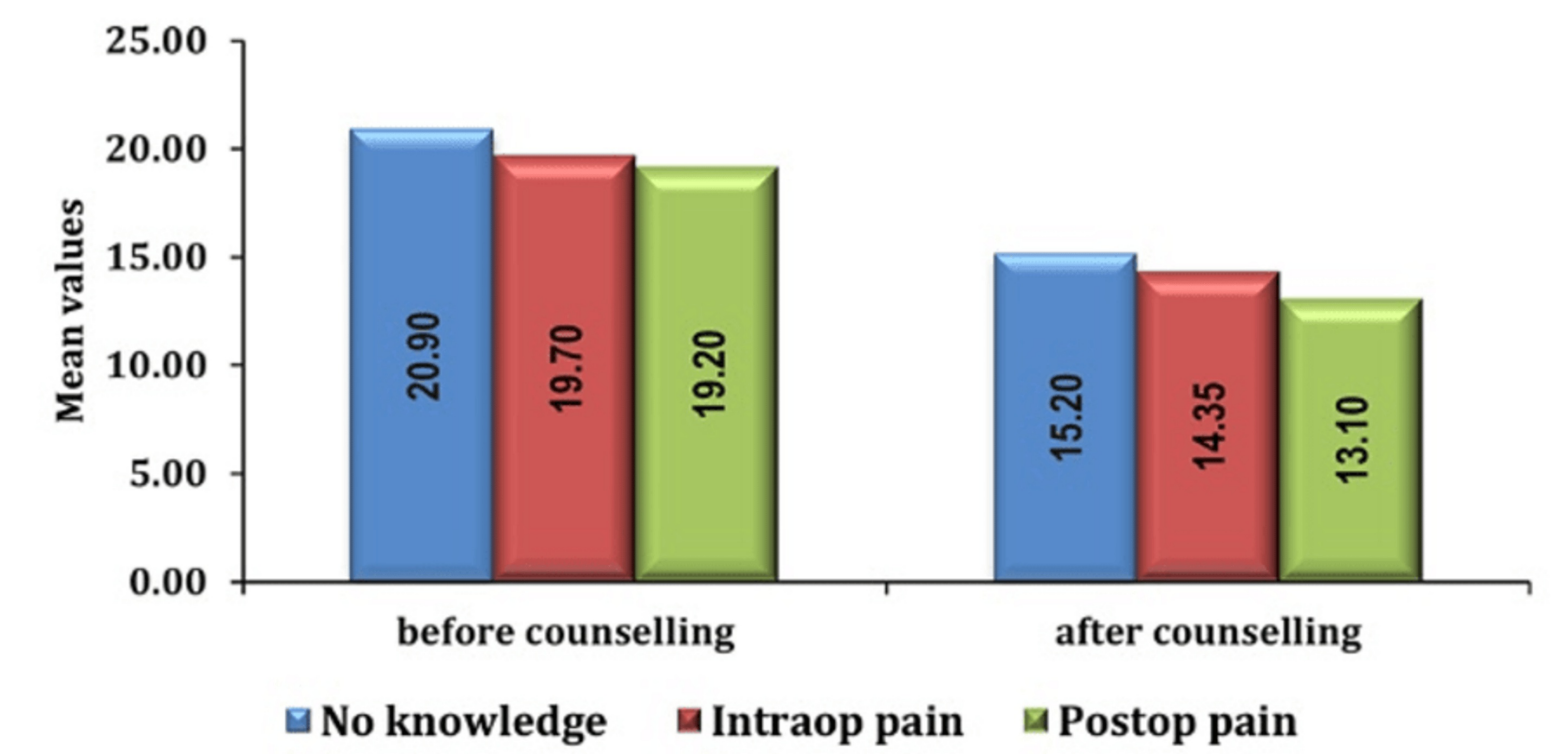
Association of anxiety score and cause of fear in group AIS pre- and post-counseling AIS: anesthesia information sheet

**Figure 10:**
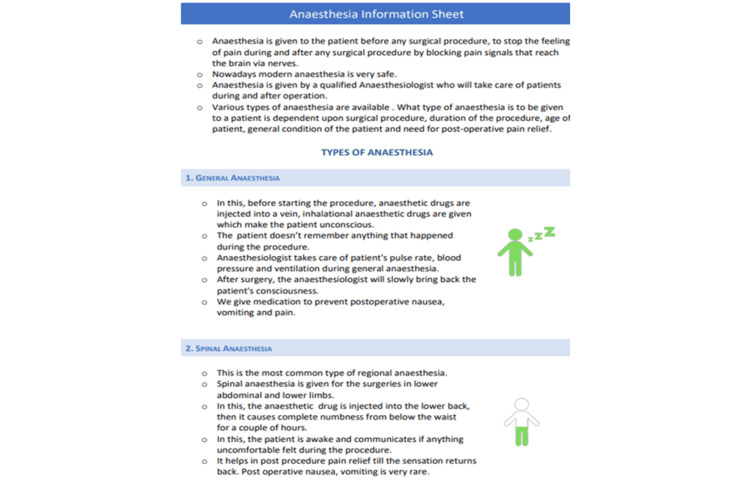
Anesthesia information sheet (part-1)

**Figure 11:**
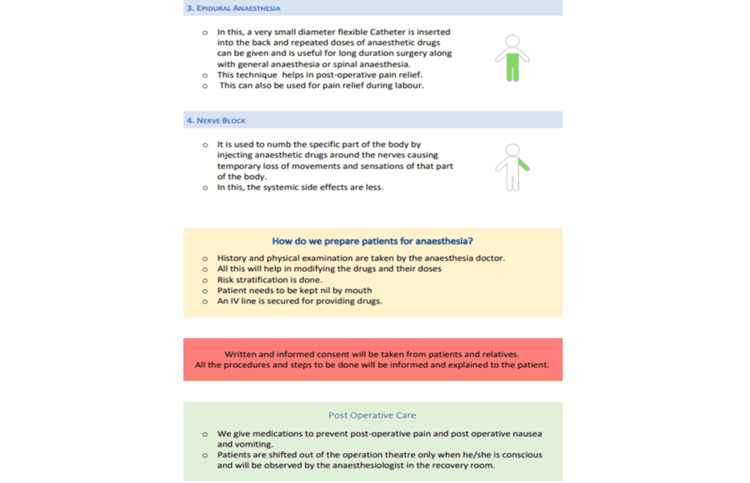
Anesthesia information sheet (part-2)

Furthermore, our investigation into the underlying causes of preoperative anxiety revealed significant findings. The most prominent contributing factor was identified as a lack of knowledge about anesthesia or surgery, followed by concerns about intraoperative and postoperative pain as seen in Figures 8, 9.

## Discussion

A statistically significant difference in anxiety scores was observed between the PC and AIS groups following counseling (p<0.05). Specifically, the post-counseling anxiety score in the PC group was 16.27±4.57, which was significantly higher than that in the AIS group, with an anxiety score of 14.25±2.42 (p=0.016). Patient anxiety levels decreased following preoperative counseling, whether through conventional verbal means or the innovative approach of utilizing an AIS. Nevertheless, employing the AIS for preoperative counseling exhibited a marked reduction in anxiety. Bansal et al. highlighted that preoperative anxiety is a neglected issue, and it must be assessed routinely during preoperative anesthesia check-ups and counseling should be undertaken by anesthesiologists with patients exhibiting heightened anxiety [8]. Preoperative anxiety can be measured using various methods, each with its strengths and limitations. Some common approaches can be self-report questionnaires, physiological measures, observational scales, psychological interviews, and combination approaches.

In medical counseling, particularly in the context of anesthesia, incorporating visual aids such as pictures alongside text has been shown to significantly enhance patient comprehension, retention, and overall experience. Visual learning plays a crucial role in demystifying complex medical concepts, making procedures and potential side effects more accessible and easier to understand for many patients. This approach aids in clarifying medical procedures, such as anesthesia and surgery, by visually depicting what will occur, thereby reducing confusion and anxiety. Furthermore, combining visual aids with text serves as an effective memory aid, improving the retention of critical information, and engaging patients more thoroughly compared to text alone. Visual content can help manage patient expectations by illustrating the equipment and process involved, which can alleviate fear and anxiety through a clearer understanding of what to expect. Additionally, visual aids bridge language barriers and simplify complex medical terms making information more accessible to non-native speakers and individuals with varying literacy levels. Overall, verbal anesthesia counseling that integrates pictures and text offers a comprehensive, engaging, and accessible communication approach, leading to improved patient understanding, reduced anxiety, and better healthcare outcomes.

When selecting a measurement method, it is essential to consider factors such as the population being studied, the specific goals of the research, and the resources available for data collection and analysis. Additionally, researchers should ensure that their chosen measures have been validated and are appropriate for use in the context of preoperative anxiety assessment. The HAM-A is a widely used and well-established instrument for measuring the severity of anxiety symptoms in adults. It was developed by Max Hamilton in 1959 and has since become one of the most commonly used scales in clinical and research settings [9]. The HAM-A is a standardized assessment tool comprising 14 items designed to evaluate a spectrum of anxiety symptoms, encompassing psychological, somatic, and behavioral manifestations. Each item is scored on a scale of zero to four or zero to two, depending on symptom severity, with higher scores indicating more pronounced anxiety symptoms. The total score range is from zero to 56, providing a quantitative measure of anxiety severity. Symptoms assessed include tension, nervousness, worry, insomnia, and somatic manifestations such as gastrointestinal and cardiovascular symptoms. This scale facilitates a comprehensive evaluation of anxiety symptomatology and enables monitoring of symptom changes over time or in response to therapeutic interventions. Typically administered via structured interviews conducted by trained clinicians, the HAM-A is widely utilized in clinical trials and research settings to assess treatment efficacy for anxiety disorders and to evaluate the impact of anxiety on functional outcomes. Its systematic approach has significantly advanced our understanding and management of anxiety disorders in adult populations, underscoring its critical role in clinical practice and research endeavors.

According to a study by W.T Kassahun et al., the distribution of anxiety status by gender showed that 53.5% of the women (129 of 185) had anxiety compared to 46.5% of the men (112 of 215), and there were more women in the anxiety group (129, 53.5%) than in the no anxiety group (56, 35.2%); the results were similar to the results in this study [10]. Women often experience higher preoperative anxiety than men due to various factors [11-13]. Hormonal variations during the menstrual cycle and menopause exert significant influence on mood and anxiety levels among women. These physiological changes, compounded by societal pressures, caregiving responsibilities, and body image concerns, contribute to a heightened psychological burden. Negative healthcare encounters, varying coping mechanisms, and distinct communication patterns further exacerbate anxiety levels. Moreover, women's perceptions regarding surgery and anesthesia reliance on social support networks and apprehensions regarding potential complications or postoperative pain amplify their overall anxiety in medical settings. Understanding these multifaceted factors is crucial for addressing and managing anxiety effectively in female patients undergoing surgical interventions.

According to a study by Maytinee et al., the intervals before and after major surgery is a high-risk period for older adults; in this setting, anxiety and depression are common and serious problems [14]. Prevalence estimates of clinically significant anxiety and depression range from 5% to 45% for anxiety. Several factors contribute to heightened preoperative anxiety among older adults compared to younger cohorts. First, older individuals often contend with multiple comorbidities, increasing concerns about potential surgical complications, and adverse outcomes. Second, age-related cognitive changes, such as slowed cognitive processing, may hinder their comprehension of surgical procedures, intensifying anxiety levels. Third, perceptions of physical frailty and vulnerability during surgery contribute to apprehensions regarding surgical outcomes. Finally, feelings of social isolation or inadequate support networks may exacerbate anxiety levels, compounding their overall preoperative distress. Recognizing and addressing these specific concerns are essential for optimizing perioperative care and improving surgical experiences among older patients.

The ASA physical status classification system plays a pivotal role in determining preoperative anxiety levels among patients. It is expected that individuals with higher ASA physical status classifications, indicative of greater comorbidities, and medical complexity experience heightened anxiety compared to those with lower ASA classifications. Heightened anxiety is likely influenced by a combination of factors, including concerns related to existing health conditions, uncertainties surrounding surgical outcomes, and feelings of diminished control over the surgical process. Understanding these dynamics is crucial for tailoring perioperative care strategies to effectively manage anxiety and improve overall patient outcomes in individuals with significant medical comorbidities.

Furthermore, it was seen that the majority of patients reporting preoperative anxiety were anxious because of surgery and anesthesia. These findings are consistent with the results of McKenzie et al., who found that in 23%, 27%, and 38% of patients, anesthesia, surgery, or a combination of the two, respectively, were the principal causes of concern [15]. Preoperative anxiety, a common phenomenon preceding surgical procedures, arises from diverse sources. Uncertainty regarding the surgical procedure and its outcomes fosters fear of the unknown among patients. Anticipation of perioperative pain intensifies anxiety levels compounded by concerns over potential adverse effects of anesthesia, including loss of consciousness and allergic reactions. Insufficient information provision and inadequate support from healthcare providers and social networks leave individuals feeling ill-prepared and isolated, further exacerbating preoperative anxiety. Recognizing and addressing these multifaceted contributors are essential for comprehensive preoperative care and optimizing patient experience and outcomes.

## Conclusions

The study suggests that combining preoperative verbal counseling with AISs is more effective in reducing patients' preoperative anxiety than verbal counseling alone. The detailed information provided by the sheets helps alleviate uncertainty and fear by clarifying what to expect during anesthesia. Integrating these sheets into preoperative counseling protocols is a valuable strategy for optimizing patient care and reducing preoperative anxiety among surgical patients.
